# Silvestrol Inhibits Chikungunya Virus Replication

**DOI:** 10.3390/v10110592

**Published:** 2018-10-30

**Authors:** Lisa Henss, Tatjana Scholz, Arnold Grünweller, Barbara S. Schnierle

**Affiliations:** 1 Paul-Ehrlich-Institut, Department of Virology, 63225 Langen, Germany; lisa.henss@pei.de (L.H.); tatjana.scholz@pei.de (T.S.); 2 Institut für Pharmazeutische Chemie, Philipps-Universität Marburg, 35032 Marburg, Germany; arnold.gruenweller@staff.uni-marburg.de

**Keywords:** chikungunya virus, silvestrol, translation, eIF4A, antiviral

## Abstract

Silvestrol, a natural compound that is isolated from plants of the genus *Aglaia*, is a specific inhibitor of the RNA helicase eIF4A, which unwinds RNA secondary structures in 5′-untranslated regions (UTRs) of mRNAs and allows translation. Silvestrol has a broad antiviral activity against multiple RNA virus families. Here, we show that silvestrol inhibits the replication of chikungunya virus (CHIKV), a positive single-stranded RNA virus. Silvestrol delayed the protein synthesis of non-structural (nsPs) and structural proteins, resulting in a delayed innate response to CHIKV infection. Interferon-α induced STAT1 phosphorylation was not inhibited nor did eIF2α become phosphorylated 16 h post infection in the presence of silvestrol. In addition, the host protein shut-off induced by CHIKV infection was decreased in silvestrol-treated cells. Silvestrol acts by limiting the amount of nsPs, and thereby reducing CHIKV RNA replication. From our results, we propose that inhibition of the host helicase eIF4A might have potential as a therapeutic strategy to treat CHIKV infections.

## 1. Introduction

Infection with chikungunya virus (CHIKV) causes chikungunya fever (CHIKF) in humans, which is a febrile illness with high fever, rash, headache, and a high rate of acute and chronic, long-lasting polyarthralgia [1]. The spread of CHIKV by mosquitos was rapidly accelerated after its vector use extended from *Aedes aegypti* to *Aedes albopictus* mosquitoes [2]. *Aedes aegypti* is confined to the tropics and sub-tropics, whereas *Aedes albopictus* also occurs in temperate and even cold temperate climate zones in Africa, Europe, and the Americas. Since its first description in 1953, CHIKV has been identified in nearly 60 countries worldwide [3]. No vaccine against or treatment for CHIKV infection is available.

CHIKV belongs to the family of Togaviridae, genus *alphavirus*. Its genome is a single-stranded, positive-sense mRNA of approximately 12 kb that contains two open reading frames encoding either the non-structural or the structural genes. Upon infection, the genomic mRNA (gmRNA) is translated into a non-structural precursor protein, which is processed by viral and cellular proteases. Non-structural proteins (nsPs) are required for viral RNA replication, which includes the synthesis of a minus-sense RNA from the gmRNA, resulting in double-stranded (ds) RNA intermediates. These intermediates can activate dsRNA-dependent protein kinase (PKR) that phosphorylates and inactivates the translation initiation factor 2 alpha (eIF2α) [4]. The minus-sense RNA serves as a template for the synthesis of subgenomic mRNA (sgmRNA) and the gmRNA. The structural proteins are translated from the sgmRNA in an eIF2α-independent manner, whereas the synthesis of nsPs requires active eIF2α [5]. However, minus-strand RNA synthesis is limited to 3–4 h post infection (hpi) and is undetectable later, when only positive-sense mRNA is synthesized. 

Alphaviruses interfere with cellular gene expression via the nsP2 protein. This has been described to inhibit transcription by the degradation of a subunit of the cellular DNA-directed RNA polymerase II (RPB1) and thereby facilitate host protein shut-off [6]. In addition, nsP2 inhibits STAT1 phosphorylation after type I interferon (IFN) treatment. CHIKV-infected cells are resistant to interferon treatment, because nsP2 inhibits STAT1 and interferon-stimulated gene expression. IFN treatment prior to infection severely reduces viral replication because interferon-stimulated genes are expressed and the innate antiviral response pathways are active [7,8].

The natural compound silvestrol can be isolated from the plant *Aglaia foveolata* and it belongs to the flavaglines that have a cyclopenta[b]benzofuran skeleton in common [9]. Silvestrol has been identified as a specific inhibitor of the DEAD-box helicase eIF4A, which is part of the translation initiation complex that binds the 5′-cap structure as the heterotrimeric initiation factor 4F (eIF4F). This is composed of the cap-binding factor eIF4E, eIF4A, and the scaffolding protein eIF4G [10,11]. The helicase eIF4A unwinds secondary structures at the 5′-UTRs of mRNAs to enable binding of the ribosomal pre-initiation complex (43S PIC). Silvestrol acts by stalling the eIF4A helicase to its mRNA substrate, depleting it from the initiation complex eIF4F, and thereby inhibiting translation [12,13]. Silvestrol has demonstrated potent anti-tumor activity in vitro and in preclinical mouse models with only minor toxicity [14,15,16,17]. Mechanistically, this was attributed to the translational inhibition of short-lived oncogenes with structured 5′-UTRs through eIF4A inactivation [18]. Moreover, antiviral activity against Ebola, corona, picorna, Zika, and hepatitis E virus has recently been described for silvestrol [19,20,21,22]. In these cases, silvestrol inhibited the translation of viral mRNAs and thereby virus replication. 

As positive-sense RNA viruses, alphaviruses translate their genes either from the gmRNA or the sgmRNA. Both mRNAs are 5′-capped and 3′-polyadenylated and they contain 5′-UTRs. The predicted RNA structures in these 5′-UTRs indicate eIF4A dependency, implying a potential inhibitory effect of silvestrol on CHIKV translation. We first confirmed the inhibitory effect of silvestrol on CHIKV replication and validated this inhibition by analyzing the viral protein expression and their consequences on innate cellular responses against the CHIKV infection.

## 2. Materials and Methods

### 2.1. Cells, CHIKV, and Reagents

All cells used for this study were cultured at 37 °C under 5% CO_2_. BHK-21 (CCL-10), HEK 293T (CRL-1573), and NIH3T3 (CRL-1658) cells were incubated in Dulbecco’s modified Eagle’s medium (DMEM; Lonza, Verviers, Belgium). Puromycin was purchased from Sigma (Sigma, Munich, Germany; #P9620) as well as human interferon α1 (IFNα) (#SRP4596). The wild-type CHIKV used for infections was a kind gift from Matthias Niedrig (Robert-Koch-Institut, Berlin, Germany) and was amplified on BHK-21 cells [23]. Silvestrol was obtained from Medchemexpress (LLC, Princeton, NJ, USA; purity >98%) and dissolved as a 6 mM stock solution in DMSO. Working solutions were prepared in DMEM.

### 2.2. Production of CHIKV-Mcherry and Infection 

The plasmid pCHIKV-mCherry [24] was in vitro-transcribed after *Not*I linearization and RNA was transfected into BHK-21 cells using Lipofectamine® 2000 (according to the manufacturer’s protocol; Life Technologies, Carlsbad, CA, USA). After 48 h, the supernatants were harvested and used to infect BHK-21 cells for virus amplification. Supernatants from these cells were stored at −80 °C. The viral titer was determined from frozen aliquots. 

CHIKV infections were done as follows: HEK 293T cells (1.5 × 10^5^ per well) were seeded onto a 24-well plate, incubated at 37 °C for 16–24 h, and counted. Subsequently, CHIKV-mCherry [24] was added at a multiplicity of infection (MOI) of 3 or as indicated. The cells were collected after 6 h in medium, washed, and resuspended in 4% paraformaldehyde in PBS, and analyzed by flow cytometry. At least 10,000 events were acquired and analyzed with an LSRII instrument and the FACS Diva software (BD Biosciences, Heidelberg, Germany).

### 2.3. CHIKV-Luci Neutralization Assay

A replicating luciferase-tagged CHIKV (CHIKV-luci) was generated by in vitro-transcription of the plasmid pCHIKV-luci, as described previously [25], followed by transfection of the RNA into BHK-21 cells. Virus (CHIKV-luci) was harvested 48 h later from supernatants and was amplified on BHK-21 cells. Cell supernatants containing virus were stored at −80 °C. Neutralization assays were performed using CHIKV-luci with the addition of silvestrol. After 6 h, 10 µL of BriteLite (PerkinElmer, Rodgau, Germany) substrate was added to the cells and incubated for 5 min at room temperature. Detection of the luciferase signal was performed while using a PHERAstar FS microplate reader (BMG LABTECH, Ortenberg, Germany). Untreated infection were expected to produce 30,000 relative light units (RLU), corresponding to an MOI of 0.06.

### 2.4. Cell Toxicity Assay

Cell toxicity of silvestrol was determined using the ATPlite 1step Luminescence Assay System from Perkin Elmer (Rodgau, Germany). Cells were incubated with silvestrol at different dilutions for 6, 24, or 48 h. By adding 7.5 µL ATPlite-substrate per well, followed by detection of the light signal with a PHERAstar FS microplate reader (BMG LABTECH, Ortenberg, Germany). The data are given as percent viability of solvent (DMSO)-treated cells.

### 2.5. SDS-PAGE and Western Blot Analysis

Proteins were first separated by SDS-PAGE and then transferred onto PVDF membranes with a Bio-Rad semi-dry blotter. The following conditions were used: 50 mM sodium borate pH 9.0, 20% methanol, and 0.1% SDS buffer at 100 mA per membrane for 75 min. After the blotting, membranes were blocked with Roti-Block™ (Carl Roth, Karlsruhe, Germany), and specific proteins were detected with antibodies directed against: CHIKV-E2 (Eurogentec, Köln, Germany, custom made), STAT1 (Sigma, Munich, Germany; #HPA000982), phospho-STAT1 (Cell Signaling, Frankfurt am Main, Germany; #7649), eIF2α (R&D Systems, Abingdon, UK; #AF3997), phospho-eIF2α (R&D Systems, Abingdon, UK; #MAB39971), mCherry (Abcam, Cambridge, UK, #ab183628), CHIKV-nsP2 (Abgenex, Bhubaneswar, India; clone ABM3F3.2E10), or β-actin (Sigma, Munich, Germany; #A5441). Detection was performed with the ECL system (Amersham, Freiburg, Germany).

### 2.6. Puromycin-Labeling

Protein synthesis was analyzed by measuring the incorporation of puromycin into growing polypeptide chains [26]. In short, 5 μg/mL puromycin was added to cells for 30 min before harvest. The cells were lysed and proteins were separated by SDS-PAGE and blotted onto PVDF membranes. Proteins were detected with a puromycin-specific antibody (Merck Millipore, Molsheim, France, #MABE343), as described above.

### 2.7. Statistical Analysis

Statistical analyses were carried out using the GraphPad Prism 5.04 software (La Jolla, CA, USA).

## 3. Results

### 3.1. Silvestrol Inhibits CHIKV Replication

**Figure 1:**
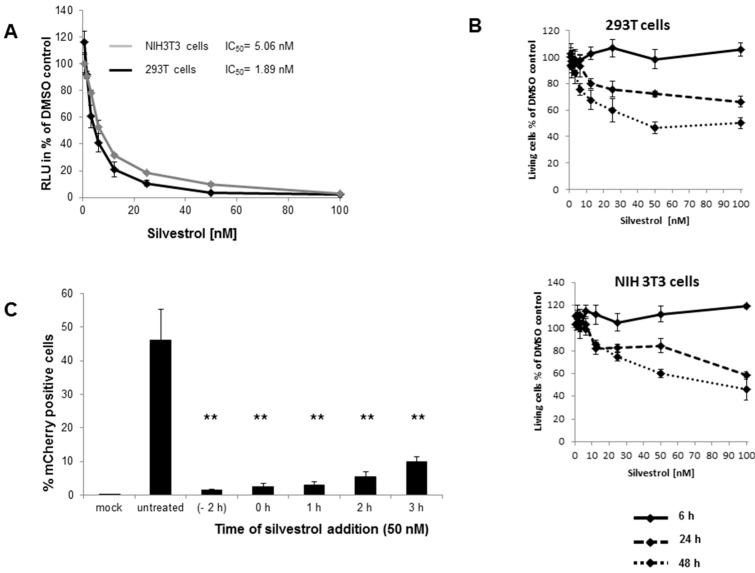
Silvestrol treatment inhibits chikungunya virus (CHIKV) replication. (**A**) CHIKV-luci replication in the presence or absence of silvestrol. Silvestrol was serially diluted, incubated with CHIKV-luci (corresponding to 30,000 relative light units [RLUs] and an MOI of 0.06) for 30 min and added to 293T or NIH3T3 cells. The neutralizing activity was detected 6 hpi as RLU. The luciferase activity is shown as a percentage, relative to the untreated control. The data show mean values of three independent experiments. IC_50_ values were calculated using the GraphPad Prism 5.04 software (La Jolla, CA, USA); (**B**) Analysis of the cytotoxicity of silvestrol. 293T or NIH3T3 cells were incubated with serial dilutions of silvestrol or solvent (DMSO) alone for 6, 24, or 48 h, and then analyzed with the ATPlite 1step Luminescence Assay System (Perkin Elmer, Rodgau, Germany). Following incubation, 7.5 µL ATPlite-substrate was added per well, and the luciferase signal was detected with a PHERAstar microplate reader (BMG LABTECH, Ortenberg, Germany). The data are given as percent viability of solvent-treated cells. The data show mean values of three independent experiments; (**C**) Time of drug addition. 293T cells were seeded in 24-well plates and infected with CHIKV-mCherry using an MOI of 1. Silvestrol (50 nM) was added at the indicated time points. Viral replication was determined 16 hpi by flow cytometry detecting mCherry. The data show mean values of experiments carried out in triplicate. ** Significant inhibition as compared to untreated CHIKV infection (*p* ≤ 0.01; unpaired Student’s *t*-test).

To assess a potential effect of silvestrol on CHIKV infection, HEK 293T and NIH3T3 cells were infected with a replicating luciferase-tagged CHIKV (CHIKV-luci) in the presence of silvestrol at a low MOI of 0.06. The recombinant CHIKV-luci expresses a fusion protein containing luciferase within the CHIKV nsP3 (non-structural protein 3) sequence [27]. Viral replication was detected by luciferase assays. Figure 1A shows the average relative luciferase (RLU) values of triplicate experiments with 293T or NIH3T3 cells being infected for 6 h with CHIKV-luci in the presence of silvestrol as percent of solvent (DMSO)-treated cells. The presence of increasing doses of silvestrol drastically inhibited CHIKV infection of 293T and NIH3T3 cells, with IC_50_ values of 1.89 and 5.06 nM, respectively. In addition, cell viability was analyzed to exclude the possibility that the observations were due to cell toxicity of the compound rather than antiviral activity (Figure 1B). Cell viability was only impaired for NIH3T3 cells when the cells were incubated with higher amounts of silvestrol starting at 24 h and for 293T cells at 48 h (Figure 1B). Silvestrol treatment for 6 h did not show any cytotoxicity and it demonstrates that the inhibition of CHIKV replication is due to silvestrol treatment.

To further analyze the mechanism of viral inhibition by silvestrol, the drug was added at different time points after infection of 293T cells with CHIKV-mCherry, a CHIKV expressing a fusion protein containing mCherry within the CHIKV nsP3 sequences [24]. Infection was performed at an MOI of 1. Adding 50 nM silvestrol 2 h before, during or 1–3 h post infection significantly inhibited the infection of 293T cells as compared to the untreated CHIKV-infected cells (Figure 1C). Addition of silvestrol 2 or 3 h after infection was slightly less efficient (Figure 1C). These data imply that silvestrol interferes with early events of the viral infection cycle.

### 3.2. Silvestrol Delays CHIKV Non-Structural and Structural Protein Synthesis

**Figure 2:**
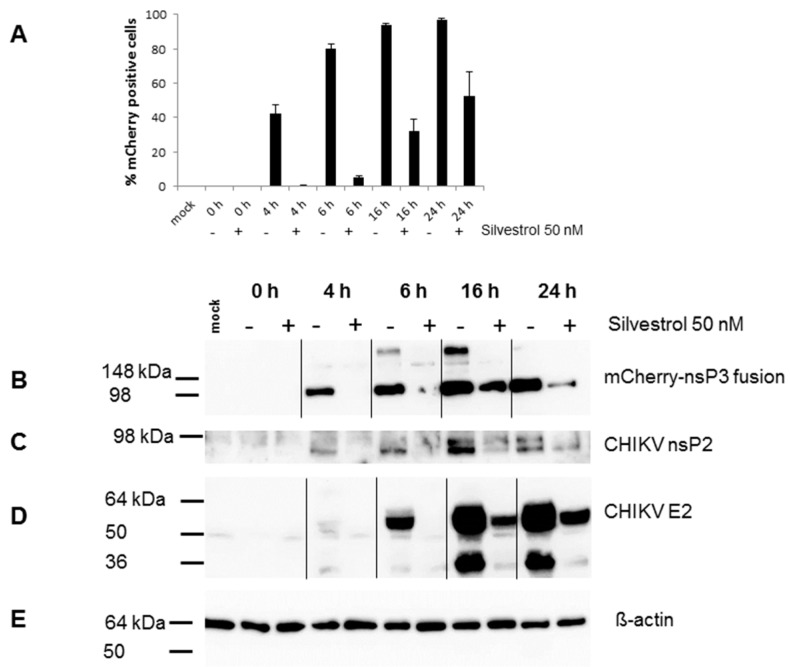
Infection of 293T cells with CHIKV-mCherry in the presence of 50 nM silvestrol. (**A**) 293T cells were seeded in 24-well plates and 50 nM silvestrol was added. Afterwards, cells were infected with CHIKV-mCherry using an MOI of 3. Viral replication was determined at the indicated time points by flow cytometry detecting mCherry. The data are mean values of three experiments carried out in triplicate. (−) Untreated cells; (+) silvestrol-treated cells; (**B**) Western blot analysis of cell lysates prepared from 293T cells infected with CHIKV-mCherry as described above. The mCherry-nsP3 fusion protein was detected with an antibody directed against mCherry and the ECL detection system (Amersham, Freiburg); (**C**) Detection of the CHIKV-nsP2 protein; and, (**D**) Detection of the CHIKV-E2 protein; (**E**) Detection of β-actin as the loading control.

Silvestrol has previously been described as an inhibitor of eIF4A that blocks translation of cellular and viral mRNAs [15,19,20,21] To investigate the effects of silvestrol on CHIKV mRNA translation, 293T cells were infected with CHIKV-mCherry at an MOI of 3 in the presence or absence of 50 nM silvestrol. Again, silvestrol treatment resulted in an inhibition of CHIKV replication, measured as the percentage of mCherry-positive cells (Figure 2A). Cell lysates were harvested at the indicated time points and analyzed by Western blot (Figure 2B–E). The non-structural proteins are synthesized from the gmRNA of the incoming virus during infection. Non-structural gene expression was analyzed by the detection of the mCherry-tagged nsP3 or nsP2 (Figure 2B). Protein expression of mCherry-nsP3 and nsP2 started early in untreated cells, at 4 hpi, and it was entirely inhibited by silvestrol treatment at that time point. However, this inhibition was transient, since mCherry-nsP3 and nsP2 were detectable in silvestrol-treated cells starting at 16 hpi, but at a much lower level than in untreated cells (Figure 2B,C). The sgmRNA, which encodes the structural proteins, is formed after RNA replication and translated at late time points of infection. The E2 protein was used as a marker for structural proteins (Figure 2D). Structural protein synthesis started in untreated cells at 6 hpi and the complete inhibition of E2 expression could be detected in infected cells treated with silvestrol (Figure 2D). This inhibition was slowly released, since E2 protein could be detected at 16–24 hpi in silvestrol-treated cells. Equal loading was confirmed by detection of β-actin (Figure 2D). These data indicate that silvestrol severely delays the translation of the gmRNA, and, since nsPs are needed for viral RNA replication, presumably inhibits RNA replication, further impeding structural protein synthesis.

### 3.3. Silvestrol Treatment Delays STAT1 Inactivation and eIF2αPhosphorylation

**Figure 3:**
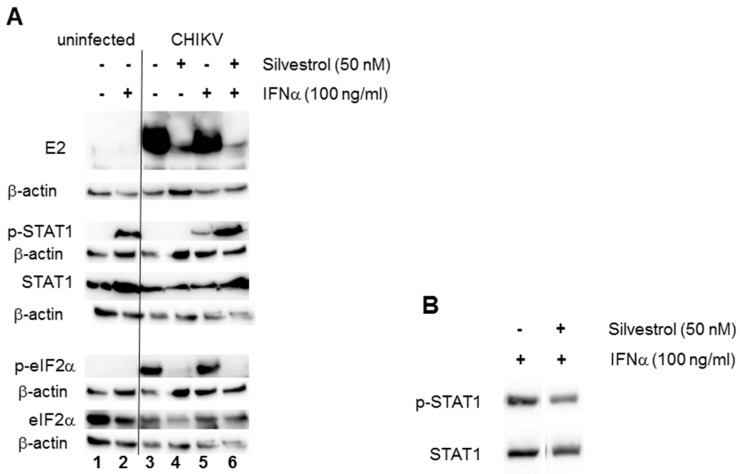
Analysis of signal transduction during CHIKV infection in the presence of 50 nM silvestrol. (**A**) HEK 293T cells were seeded in six-well plates and were infected with CHIKV using an MOI of 1, and 50 nM silvestrol was added where indicated. Cells were treated with IFNα for 30 min before harvest if indicated, and Western blot analysis of cell lysates was performed. (−) Untreated cells; (+) treated cells. In lanes 3–6, cells were infected with CHIKV. The CHIKV E2 protein, STAT1, eIF2α, and their phosphorylated proteins were detected with specific antibodies and secondary HRP-coupled antibodies, and the ECL detection system (Amersham, Freiburg). Equal loading of each blot was controlled by detection of β-actin; and, (**B**) Uninfected HEK293T cells were treated with IFNα for 30 min before harvest and either treated with silvestrol for 16 h or left untreated. Western blot analysis of cell lysates was performed and p-STAT1 and STAT1 were detected. STAT1 served as a loading control.

To obtain further insights into the consequences of silvestrol treatment during CHIKV infection, its potential role in innate immunity, particularly in interferon signaling, was analyzed. It has been previously reported that alphavirus infection leads to the activation of the interferon regulatory factor 3 (IRF3), and subsequently, the transcription of IRF-3-dependent antiviral genes, including type I interferons (IFNs) [28]. Translation of these genes could not be observed, but this may have been due to cellular protein synthesis shut-off induced by the virus [5]. To study the effect of silvestrol treatment on IFN signaling, CHIKV-infected cells were treated with IFNα and then analyzed. Again, 293T cells were infected with CHIKV at an MOI of 1 in the presence or absence of 50 nM silvestrol, followed by IFNα treatment (100 ng/mL) for 30 min before cell harvest. Cell lysates were prepared at 16 hpi. As shown in Figure 3A, E2 protein expression in infected 293T cells was again inhibited by silvestrol and IFNα treatment did not change this (Figure 3A, lanes 4 and 6). CHIKV infection alone did not induce STAT1 phosphorylation, suggesting that the amounts of type I IFN secreted during infections are not sufficient (Figure 3A, lane 3). However, IFNα treatment of cells induced STAT1 phosphorylation, which was reduced in CHIKV-infected cells as compared to uninfected cells (Figure 3, lanes 2 and 5). This is consistent with the reported ability of CHIKV-nsP2 to inhibit STAT1 phosphorylation [7]. Silvestrol treatment of the cells, however, allowed STAT1 phosphorylation (Figure 3, lane 6), presumably by restricting CHIKV protein synthesis and thereby preventing the CHIKV-induced inhibition of STAT1 signaling. IFNα and silvestrol treatment of uninfected cells for 16 h did not significantly influence STAT1 phosphorylation (Figure 3B).

As has been described previously [29], CHIKV infection induced eIF2α phosphorylation (Figure 3, lanes 3 and 5). Silvestrol treatment abrogated eIF2α phosphorylation and this phosphorylation was independent of IFNα treatment (Figure 3, lanes 4 and 6). Taken together, these results demonstrate that, by inhibiting CHIKV protein synthesis and replication, silvestrol treatment also delays the viral countermeasures against cellular antiviral response pathways in infected cells.

### 3.4. Effects of Silvestrol on Host Protein Shut-Off Induced by CHIKV Infection

**Figure 4:**
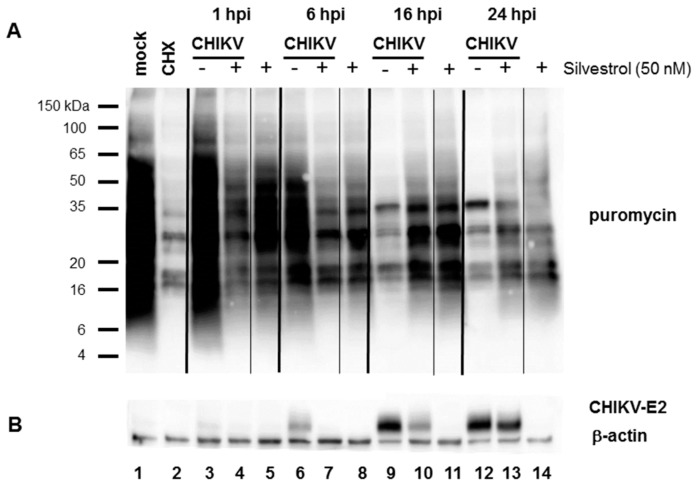
Analysis of cellular protein synthesis during CHIKV infections in the presence of 50 nM silvestrol. HEK 293T cells were infected with CHIKV at an MOI of 10 and either treated with 50 nM silvestrol (+) or left untreated (−). Cells were harvested after the indicated times of infection. Before harvest, cells were treated with 5 mg/mL puromycin for 30 min. (**A**) Detection of proteins with a puromycin-specific antibody. CHX: cycloheximide; (**B**) Detection of the CHIKV-E2 protein and β-actin as the loading control.

A hallmark of CHIKV infection is the shut-off of host protein synthesis, which is a means of preventing innate responses against the virus. To analyze the effect of silvestrol on host shut-off, total cellular protein synthesis was analyzed by measuring the incorporation of puromycin into growing polypeptide chains [26]. HEK 293T cells were infected with CHIKV wt (MOI 10) and 5 μg/mL puromycin was added to the cells for 30 min before harvest at the indicated time points. Proteins were detected by Western blot analysis with a puromycin-specific antibody. As a control, cells were treated with the translation inhibitor cycloheximide, which ablates puromycin labeling (Figure 4A, lane 2). Figure 4A shows that silvestrol treatment for 1 h had already reduced cellular protein expression (Figure 4A, lane 5). The time point 1 hpi indicates the addition of silvestrol and infection of cells with CHIKV at an MOI of 10 for 30 min, followed by incubation for another 30 min with medium containing puromycin. CHIKV infection led to a dramatic reduction in protein synthesis (Figure 4A, lanes 9 and 12), resulting in an almost complete shut-off by 16 hpi. Silvestrol treatment of infected cells, however, rescued cellular protein synthesis to a level that is similar to that observed following silvestrol treatment without CHIKV infection. Even 24 hpi, proteins could still be labeled with puromycin (Figure 4A, lane 13), whereas CHIKV infection severely blocked protein synthesis. Infection of the cells was confirmed by analyzing CHIKV E2 protein expression. Silvestrol treatment, although expected to inhibit translation, rather prevented the cellular protein shut-off by delaying CHIKV protein synthesis.

## 4. Discussion

Silvestrol is a specific and potent inhibitor of eIF4A and inhibits mRNA translation by blocking the unwinding of structured 5′-UTRs and depleting eIF4A from the eIF4F complex [11,12]. Alphaviruses have two 5′-capped and 3′-polyadenylated mRNAs that are translated, the positive-strand gmRNA that encodes the non-structural proteins (nsPs) and the sgmRNA that encodes the structural proteins. During viral replication, gmRNA is converted into minus-strand RNA with the help of nsPs. This is the template for positive-strand gmRNA and sgmRNA. The sgmRNA is transcribed from an internal promoter in the minus-strand replication intermediate and it has a conserved structured 5′-UTR [30]. The gmRNA has a short structured sequence, so both mRNAs might be eIF4A dependent and targets for silvestrol-mediated translational inhibition. 

Indeed, we observed a complete and dose-dependent block of CHIKV replication by silvestrol with an IC50 of 1.89 nM for 293T cells during infection at a low MOI. Time-of-drug-addition experiments were performed with CHIKV-mCherry, which expresses an nsP3-mCherry fusion protein allowing for CHIKV replication to be monitored by flow cytometry. Silvestrol treatment before infection, during, and 1 hpi severely reduced CHIKV replication at 16 hpi (Figure 1C). Adding silvestrol 2 or 3 hpi still inhibited CHIKV replication, however less efficiently than earlier treatment. These results indicate that silvestrol inhibits CHIKV replication, but also nsP synthesis, which is needed for viral replication. A viral growth kinetic for CHIKV-mCherry infection at a higher MOI of 3, revealed that silvestrol delays the onset of viral replication, resulting in a lower total amount of virus (Figure 2A). Western blot analysis confirmed that nsP synthesis was delayed after silvestrol treatment and could only be clearly detected at 16 hpi as compared to 4 hpi without silvestrol treatment (Figure 2). Non-structural proteins are needed for RNA replication and minus-strand RNA, gmRNA, and sgmRNA synthesis. Inhibition of structural protein synthesis by 50 nM silvestrol was also observed (Figure 2). However, it could not be determined whether silvestrol acted directly on sgmRNA translation or whether structural protein synthesis was inhibited as a result of delayed nsP synthesis. No mutations in the sgmRNA 5′-UTR in silvestrol-treated CHIKV-infected cells could be found after sequencing the viruses [31], indicating the presence of an escape mechanism involving eIF4A-independent translation or an insufficient block of nsP synthesis, which is more apparent at higher MOIs. Other RNA helicases, like DHX29 or DDX3, have been described to participate in the RNA unwinding process during translation initiation and might give rise to the observed inefficient viral protein synthesis [32,33].

As a consequence of its effect on CHIKV replication, silvestrol treatment attenuated innate sensing and antiviral responses during infections. The shut-off of host protein synthesis by the virus prevents the production of antiviral proteins, such as interferons. Translation of cellular and gmRNA is downregulated during late phases of infection, when sgmRNA synthesizes structural proteins very efficiently [34]. Translation of mRNAs also requires the initiation factor eIF2α. Activation of protein kinase R (PKR) by double-stranded viral RNA replication intermediates results in the phosphorylation of eIF2α, which thereby becomes inactivated and blocks translation [35]. The eIF2α phosphorylation induced by CHIKV infection at 16 hpi was prevented by silvestrol treatment (Figure 3). This suggests that cellular protein shut-off could be less efficient in silvestrol-treated cells. Analysis of newly synthesized total protein showed that although silvestrol treatment alone inhibited protein synthesis to a certain degree, at late time points (16 and 24 hpi) when CHIKV replication is still impaired by silvestrol treatment, host protein shut-off was less efficient (Figure 4). The alphavirus nsP2 protein has been described to be responsible for host protein shut-off by inhibiting cellular transcription [36,37,38]. Therefore, silvestrol might indirectly, by inhibiting gmRNA translation, extend the expression of host proteins by decreasing the amount of nsP2 protein or dsRNA replication intermediates.

Likewise, the STAT1 phosphorylation that is induced by IFNα treatment of 293T cells, which was inhibited in CHIKV-infected cells 16 hpi, was not inhibited in silvestrol-treated infected cells (Figure 3). Again, the CHIKV nsP2 protein has been described to be involved in this process. Like eIF4A, nsP2 is an RNA helicase and it has been shown to interfere with JAK-STAT signaling, resulting in the inhibition of STAT1 phosphorylation. Its methyltransferase-like domain has recently been found to be responsible for STAT1 blockage [7,39]. However, it is very unlikely that silvestrol inhibits the helicase activity of nsP2. The inhibition is rather expected to be due to the lower total amount of nsP2 resulting from the attenuated gmRNA translation in silvestrol-treated, infected cells.

In summary, by specifically targeting the host translation initiation factor eIF4A, silvestrol inhibits the translation of CHIKV gmRNA and thereby CHIKV replication. When infections were performed at a low MOI of 0.06, CHIKV infection was completely blocked. However, at higher MOIs, this block was leaky, most likely because alternative helicases facilitate inefficient gmRNA translation and severely delay and attenuate CHIKV replication.

High virus titers develop in vertebrates that are infected with alphavirus, which are required for their transmission to mosquitos during the blood meal. Therefore a decreased viral load in infected patients might prevent the rapid spread of alphaviruses in naïve populations. Additionally, alphaviruses are very sensitive to type I IFNs and have developed strategies to prevent IFN induction and the activation of antiviral genes. The low level of CHIKV protein synthesis and replication caused by silvestrol treatment might be able to prime uninfected cells by paracrine mechanisms, leading to the activation of antiviral genes [40]. Silvestrol treatment of infected patients might decrease CHIKV loads, which could improve the development of an effective adaptive immune response against CHIKV.
